# Supplementary material for: Molecular insights into the endoperoxide formation by Fe(II)/α-KG-dependent oxygenase NvfI
Source: Nat Commun. 2021 Jul 20;12:4417. doi: 10.1038/s41467-021-24685-6 (PMC8292354; doi:10.1038/s41467-021-24685-6)
Supplement: Supplementary file 1 — Supplementary Information [file 41467_2021_24685_MOESM1_ESM.pdf]

Table of contents

Supplementary Tables

Supplementary Table 1. Results of ^{18}O-labeling experiment.

Supplementary Table 2. Data collection and refinement statistics

Supplementary Table 3. NMR table of **5**.

Supplementary Table 4. NMR table of **6**.

Supplementary Table 5. Primer list used in this study.

Supplementary Figures

Supplementary Figure 1. Proposed reaction mechanisms of endoperoxide formation reactions.

Supplementary Figure 2. Phylogenetic analysis of NvfI homologues and other Fe(II)/α-KG dependent oxygenases.

Supplementary Figure 3. Stoichiometric analysis of α-KG and O2.

Supplementary Figure 4. The proposed reaction mechanism for the incorporation of $H_2{}^{18}O$ into **3**.

Supplementary Figure 5. Determination of the size of NvfI in the solution state.

Supplementary Figure 6. Comparison of overall structures of NvfI and oxygeneases in the biosynthesis of meroterpenoids.

Supplementary Figure 7. Stereo views of active site architectures of NvfI.

Supplementary Figure 8. Modeling of the substrate binding in open conformation of NvfI.

Supplementary Figure 9. Key HMBC, COSY, and NOESY signals of **5** and **6**.

Supplementary Figure 10. Comparison of the active site of NvfI wild type and W199F variant.

Supplementary Figure 11. Time course enzyme reaction and Michaelis-Menten plots of the NvfI and NvfI-Y116A kinetics.

Supplementary Figure 12. The active site shapes of NvfI wild type and H138A variant.

Supplementary Figure 13. The reaction of NvfI and reaction mechanism for the formation of **1**.

Supplementary Figure 14-25. NMR spectra for synthetic substrates.

Supplementary Tables

Supplementary Table 1. Results of ^{18}O-labeling experiment (n = 3). Please also see Figure 2.

	m/z = 495	m/z = 497	m/z = 499	m/z = 501	m/z = 503
$H_2O/^{18}O_2$ (%)	2.0 ± 0.3	1.1 ± 0.3	95.7 ± 1.1	1.2 ± 0.6	-
$H_2{}^{18}O/O_2$ (%)	17.0 ± 3.2	62.5 ± 4.5	20.4 ± 1.4	-	-
$H_2{}^{18}O/^{18}O_2$ (%)	0.4 ± 0.05	1.4 ± 0.5	18.3 ± 0.4	56.7 ± 0.9	23.2 ± 0.9

Supplementary Table 2. Data collection and refinement statistics.

	NvfI_Zn	NvfI_αKG_**2**	NvfI_NOG_**2**	NvfI_W199F_NOG_**2**
**Data collection**				
Space group	C2	C2	$P2_12_12_1$	$P2_12_12_1$
Cell dimensions				
a, *b*, *c* (Å)	157.3, 48.2, 79.5	157.3, 48.3, 79.6	47.8, 79.3, 154.5	47.9, 81.1, 151.8
α, β, γ (°)	101.3	101.4	90	90
Resolution (Å)	50.2-1.9	46.1-1.9	47.8-2.3	47.9-2.3
	(1.97-1.92)	(1.94-1.90)	(2.38-2.30)	(2.38-2.30)
R_{merge} (%)	7.0 (52.7)	14.1 (84.3)	17.8 (91.0)	13.6 (72.1)
$I / \sigma I$	29.0 (5.5)	11.1 (3.0)	8.7 (2.1)	12.2 (3.0)
Completeness (%)	99.8 (97.2)	100 (100)	100 (100)	100 (100)
Redundancy	13.8 (13.3)	7.4 (7.5)	7.6 (7.9)	7.5 (7.9)
**Refinement**				
Resolution (Å)		39.1-1.9	45.6-2.3	45.7-2.3
No. reflections		46668	26881	27083
R_{work} / R_{free}		17.3/19.3	18.3/23.5	19.7/24.8
No. atoms				
Protein		4419	4436	4391
Ligand/ion		54	54	54
Water		421	276	261
B-factors				
Protein		24.8	30.0	29.4
Ligand/ion		22.5	23.4	30.3
Water		33.2	31.9	31.4
R.m.s. deviations				
Bond lengths (Å)		0.009	0.004	0.003
Bond angles (°)		1.241	0.685	0.575

*Data was collected from one crystal. *Values in parentheses are for highest-resolution shell.

Supplementary Table 3. NMR table of **5**

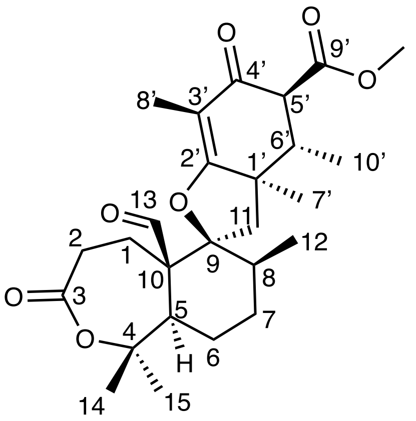

	^{13}C	^{1}H			HMBC
position	δ (ppm)	δ (ppm)	intensity	multiplicity	
1	26.9	2.69	1H	m	2
		1.60	1H	m	
2	32.8	2.69	1H		3, 10
		2.45	1H	dd (13.3, 5.6 Hz)	
3	176.1				
4	85.9				
5	45.6	2.38	1H	m	4, 6, 10, 13, 14
6	22.4	2.39	1H	m	5, 8, 10
		1.78	1H	m	
7	30.0	1.68	1H, overlap	m	6
		2.00	1H	m	
8	38.2	2.00	1H, overlap	m	7
9	100.7				
10	58.3				
11	42.0	2.15	1H	m	8, 9, 1', 2', 6', 7'
		2.47	1H	m	
12	16.8	0.94	3H	d (5.0 Hz)	7, 8, 9
13	203.8	10.22	1H	s	1, 10
14	26.6	1.46	3H	s	4, 5, 15
15	30.8	1.63	3H	s	4, 5, 14
1’	45.4				
2’	184.1				
3’	108.9				
4'	195.5				
5'	59.2	3.31	1H	m	4', 6', 9', 10'
6'	41.9	2.46	1H	m	1', 5', 10'
7'	21.9	1.09	3H	s	11, 1', 2', 6'
8'	7.8	1.66	3H	s	2', 3', 4'
9'	173.0				
10'	14.5	0.99	3H	d (5.1 Hz)	1', 5', 6'
11'	52.5	2.71	3H	s	9'

Supplementary Table 4. NMR table of **6**

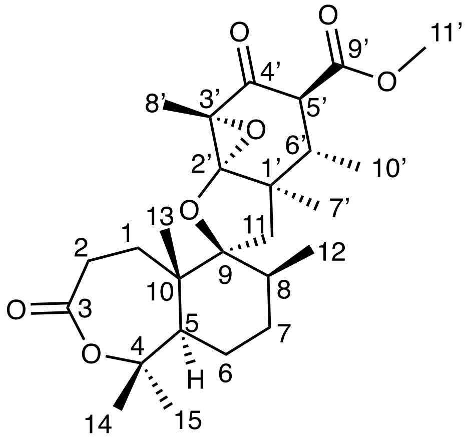

	^{13}C	^{1}H			HMBC
position	δ (ppm)	δ (ppm)	intensity	multiplicity	
1	35.4	1.84	1H	m	2, 9, 10
		1.75	1H, overlap	m	
2	32.1	2.86	1H		1, 10
		2.50	1H	dd (13.3, 5.6 Hz)	
3	176.5				
4	85.5				
5	47.5	2.06	1H	m	4, 6, 13, 15
6	22.0	1.20	1H	m	4, 5, 7, 8
		1.75	1H, oevrlap	m	
7	25.6	1.75	2H, overlap	m	6, 8
8	29.1	1.75	1H, overlap	m	7, 9, 12
9	93.3				
10	36.4				
11	41.6	1.63	1H	d (13.4 Hz)	9. 7'
		1.75	1H, overlap	m	
12	16.4	0.93	3H	d (6.0 Hz)	7, 9
13	31.8	1.27	3H	s	1, 5, 9, 10
14	26.0	1.42	3H	s	4, 5, 15
15	28.0	1.51	3H	s	4, 5, 14
1’	46.8				
2’	94.3				
3’	77.0				
4'	207.0				
5'	56.5	3.69	1H	d (12.9 Hz)	4', 6', 9', 10'
6'	42.4	2.31	1H	dq (13.4, 6.7 Hz)	11, 1', 5', 7', 10'
7'	14.8	1.26	3H	s	11, 1', 2', 6'
8'	7.8	1.29	3H	s	2', 3', 4'
9'	169.0				
10'	12.3	0.90	3H	d (6.8 Hz)	1', 5', 6'
11'	51.6	3.72	3H	s	

Supplementary Table 5. Primers used in this study.

S114A_f	GACCATGTCGTCTGTTTCGCCCCCTACACTCGCAAGG
S114A_r	CCTTGCGAGTGTAGGGGGCGAAACAGACGACATGGTC
Y116A_f	GTCGTCTGTTTCAGCCCCGCCACTCGCAAGGAGAAC
Y116A_r	GTTCTCCTTGCGAGTGGCGGGGCTGAAACAGACGAC
Y116F_f	GTCGTCTGTTTCAGCCCCTTTACTCGCAAGGAGAAC
Y116F_r	GTTCTCCTTGCGAGTAAAGGGGCTGAAACAGACGAC
F127A_f	GAACTCGGAAAAGGGAATAGCCGGACAGCCTGCCAG
F127A_r	CTGGCAGGCTGTCCGGCTATTCCCTTTTCCGAGTTC
F127I_f	GAACTCGGAAAAGGGAATAATCGGACAGCCTGCCAG
F127I_r	CTGGCAGGCTGTCCGATTATTCCCTTTTCCGAGTTC
H138A_f	CCAGAACGGTCCACTGCGATGCCACTCCAGCAGC
H138A_r	GCTGCTGGAGTGGCATCGCAGTGGACCGTTCTGG
H138F_f	CAGAACGGTCCACTGCGATTTCACTCCAGCAGCAG
H138F_r	CTGCTGCTGGAGTGAAATCGCAGTGGACCGTTCTG
W199A_f	GACTTGCACCCGGTCCACGCGCTCAGGTACGAGAAG
W199A_r	CTTCTCGTACCTGAGCGCGTGGACCGGGTGCAAGTC
W199F_f	GACTTGCACCCGGTCCACTTTCTCAGGTACGAGAAG
W199F_r	CTTCTCGTACCTGAGAAAGTGGACCGGGTGCAAGTC
R201A_f	GCACCCGGTCCACTGGCTCGCGTACGAGAAGAAGG
R201A_r	CCTTCTTCTCGTACGCGAGCCAGTGGACCGGGTGC
E208A_f	CGAGAAGAAGGATACCGCACCACCCTTCCAACTC
E208A_r	GAGTTGGAAGGGTGGTGCGGTATCCTTCTTCTCG

Supplementary Figures

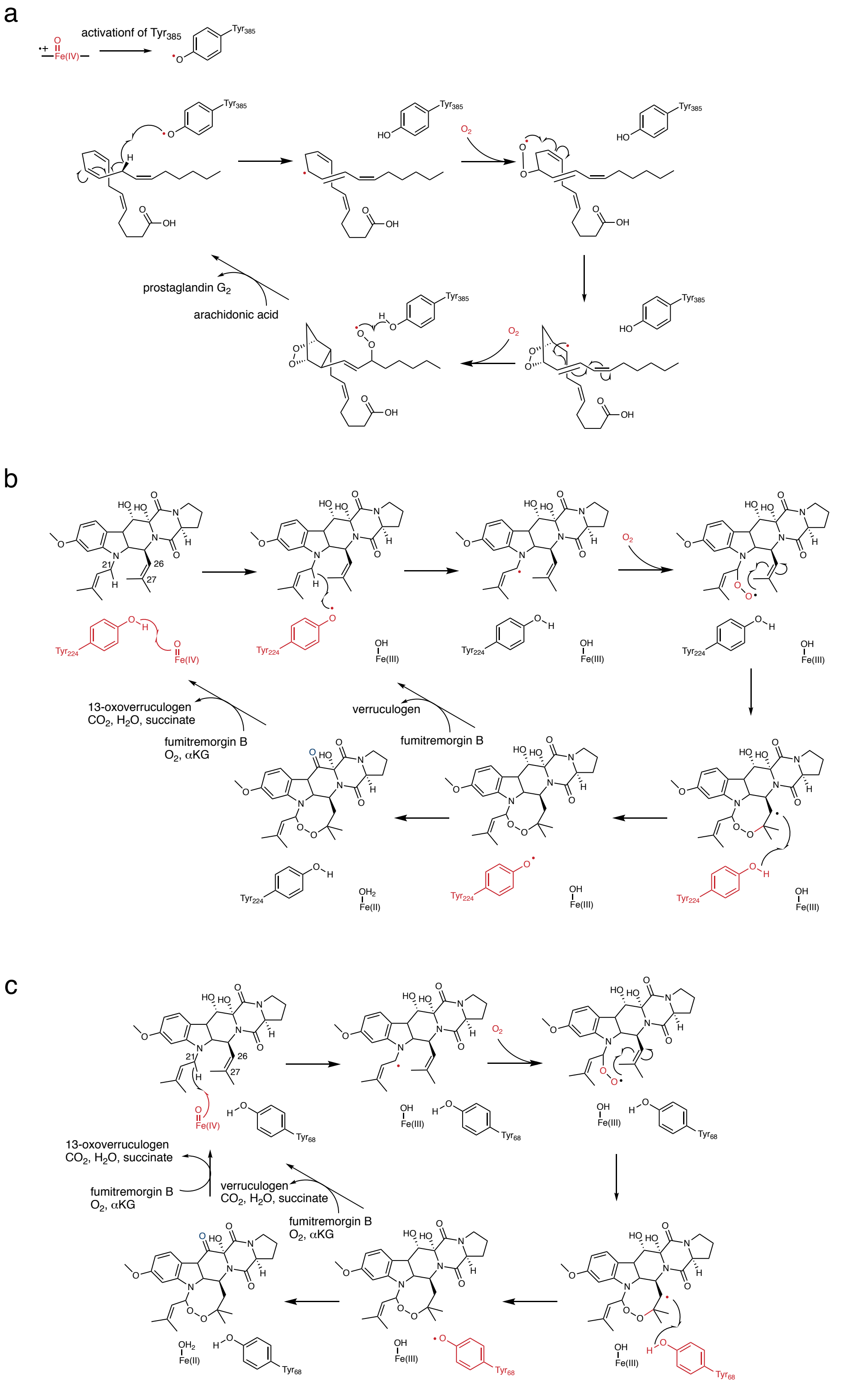

Supplementary Figure 1. Proposed mechanisms of endoperoxide formation reactions. Reaction mechanisms of a) COX, b) FtmOx1 proposed by Yan *et al.*, and c) FtmOx1 by Dunham *et al.*

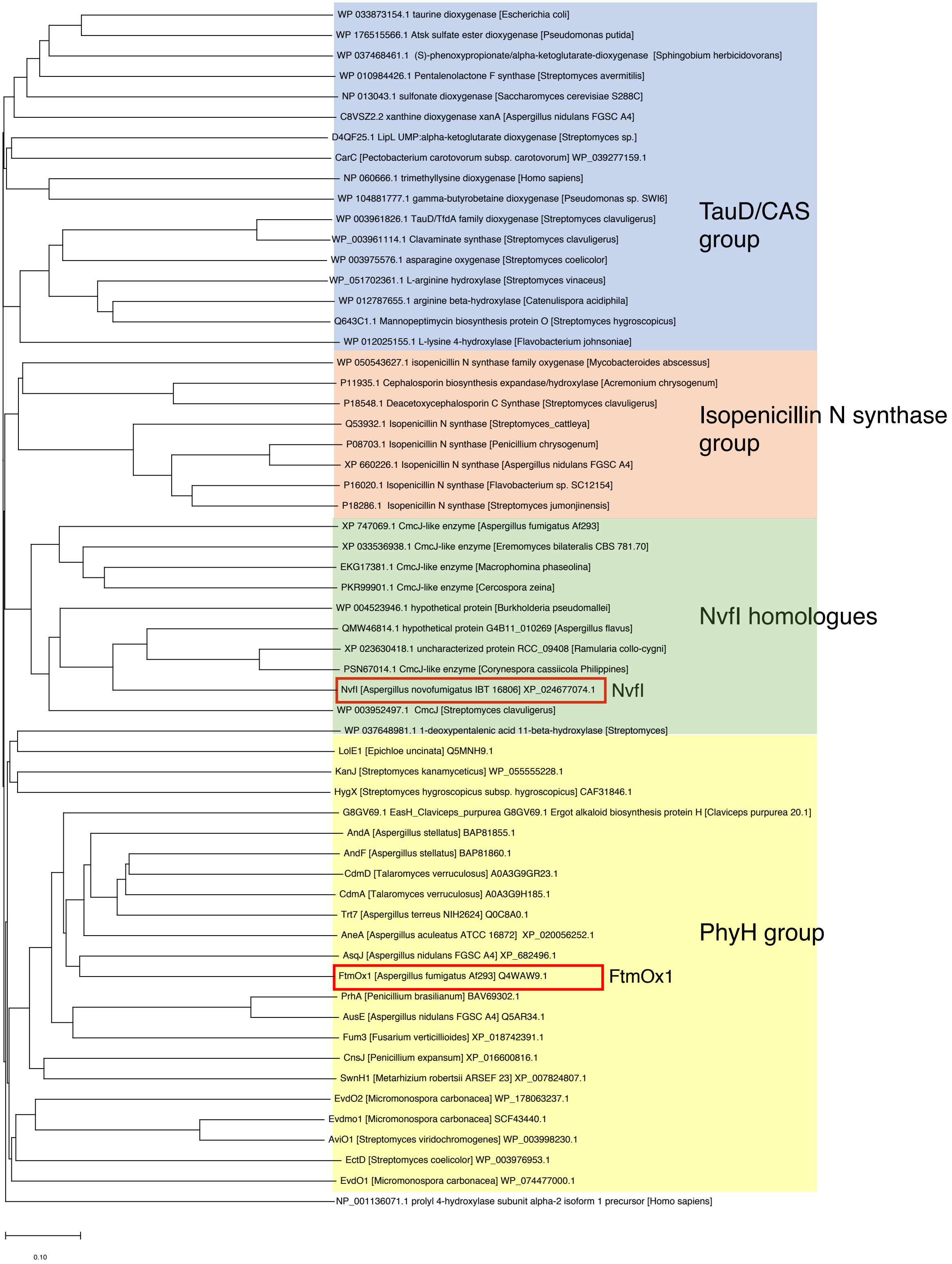

Supplementary Figure 2. Phylogenetic analysis of NvfI homologues and other Fe(II)/α-KG-dependent oxygenases. The multiple sequence alignments were performed using Clustal W 2.0.12. The neighbor-joining method (MEGAX) was used to generate this phylogenetic tree. Prolyl 4-hydroxylase from *Homo sapiens* (NP_001136071.1) was used as the outgroup.

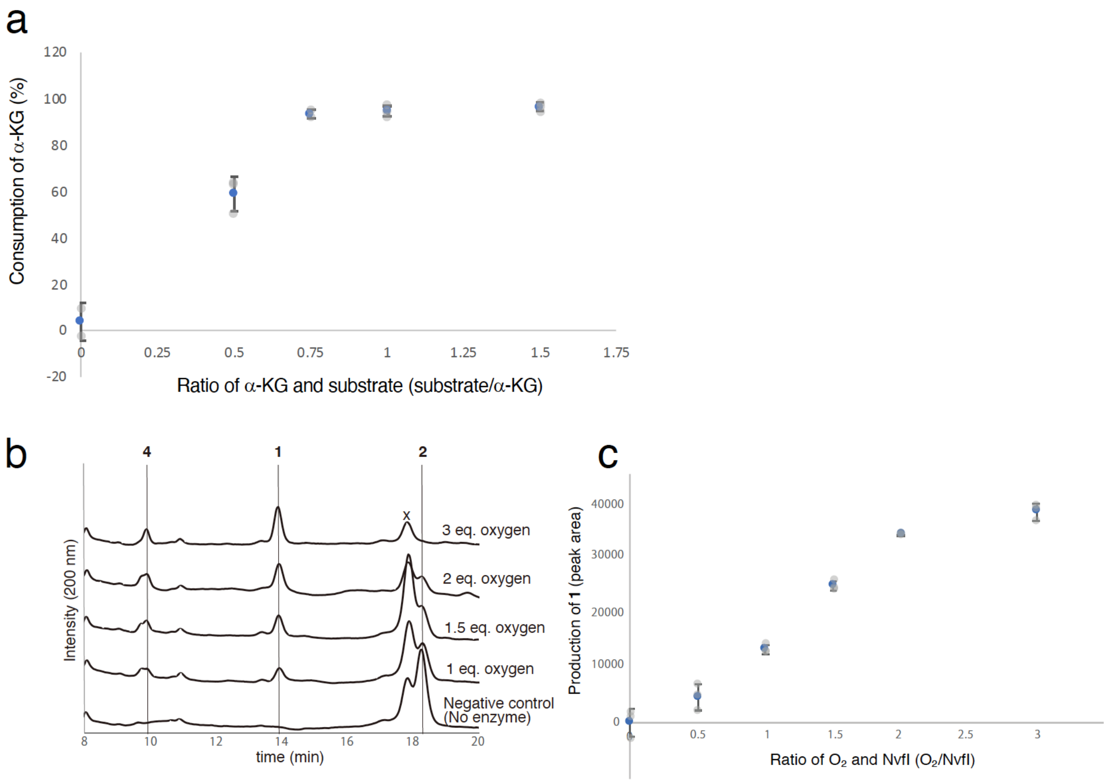

Supplementary Figure 3. Stoichiometric analysis of α-KG and O_2. (a) Consumption of α-KG in the enzyme reactions of NvfI, using varied ratios of **2** and α-KG. (b) HPLC charts of the enzyme reactions with different concentration of O_2. (c) Production of **1** in the enzyme reactions of NvfI, using various concentration of O_2. The x peak shows an impurity from the reaction buffer. All experiments were repeated independently more than three times with similar results. The blue dots are means of n = 3 independent experiments and error bars indicate standard deviations.

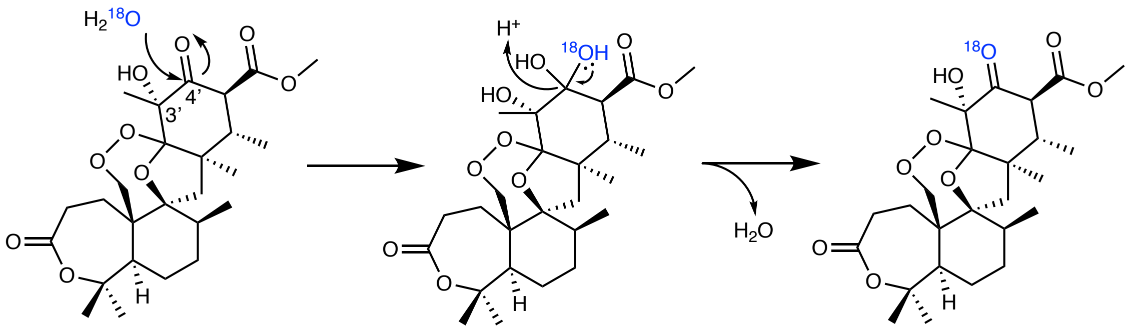

Supplementary Figure 4. The proposed reaction mechanism for the incorporation of $H_2{}^{18}O$ into **3**.

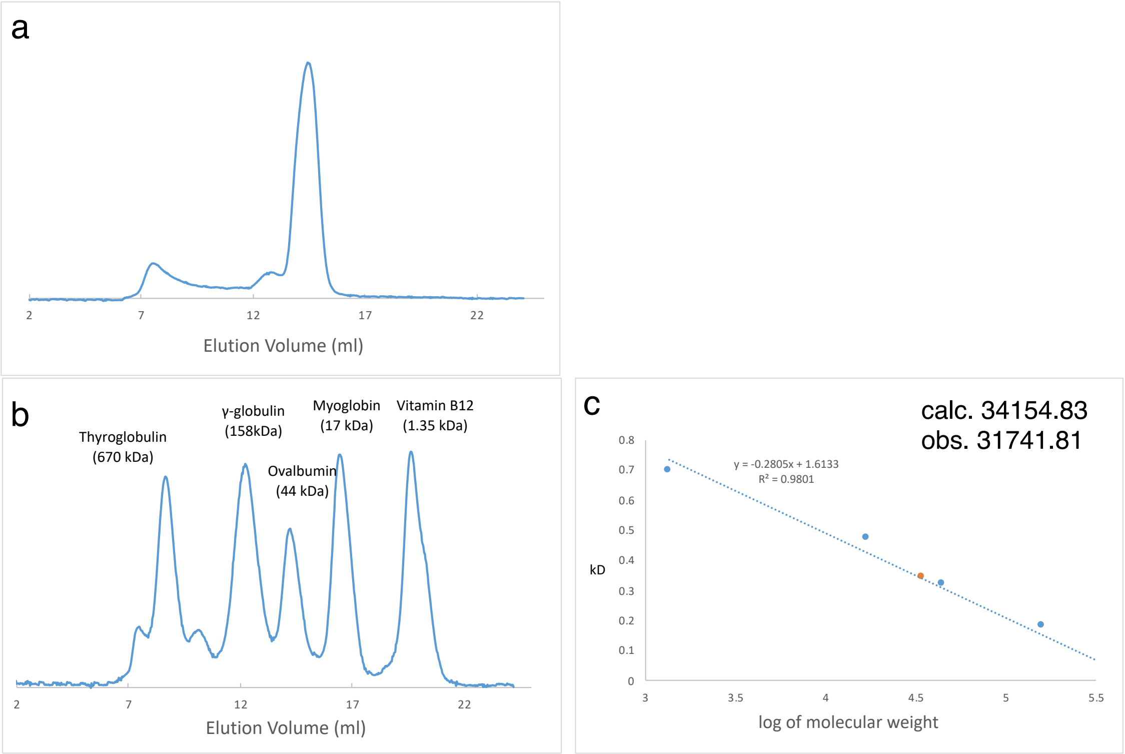

Supplementary Figure 5. Determination of the size of NvfI in the solution state. Gel-filtration analyses of a) NvfI and b) standard proteins (Bio-Rad #1511901). (c) Calibration curve prepared by plotting the KD value for each standard. The blue points and orange point show each standard protein and NvfI, respectively.

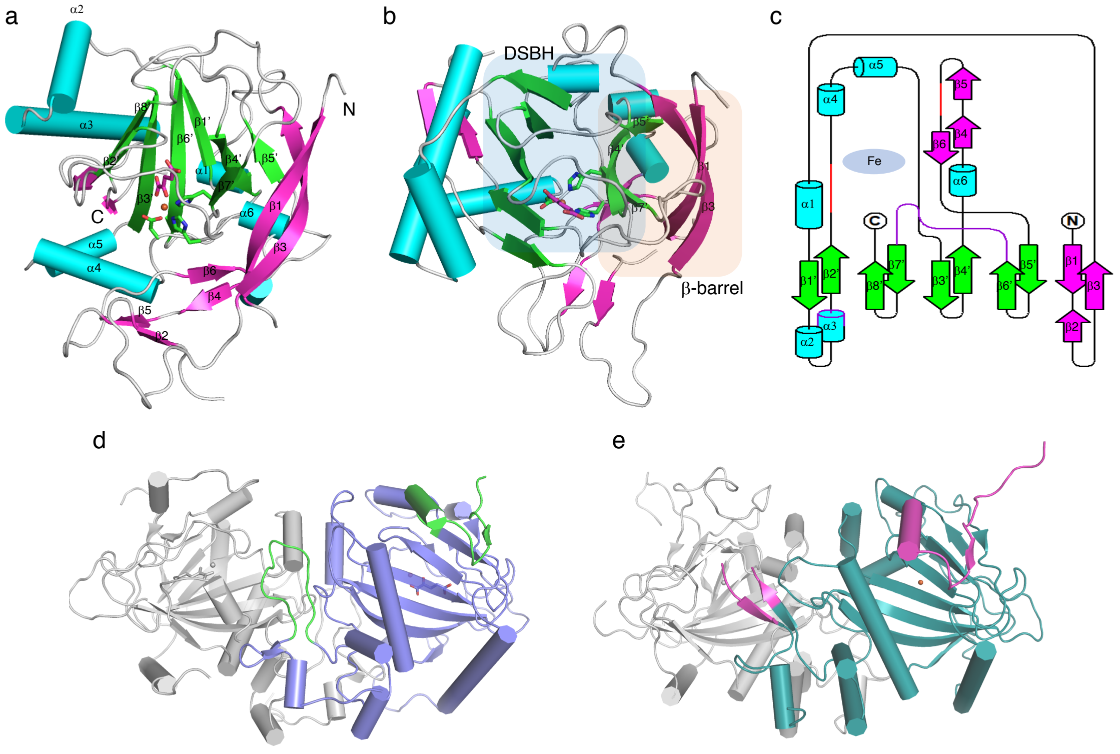

Supplementary Figure 6. Comparison of the overall structures of a-c) NvfI and d, e) dioxygenases in the biosynthesis of meroterpenoids. a, b) Overall structure of NvfI. c) Schematic depiction of the secondary structure elements of NvfI. Overall structures of d) FrmOx1 (PDB ID: 6OXH) and e) AndA (PDB ID: 5ZM4).

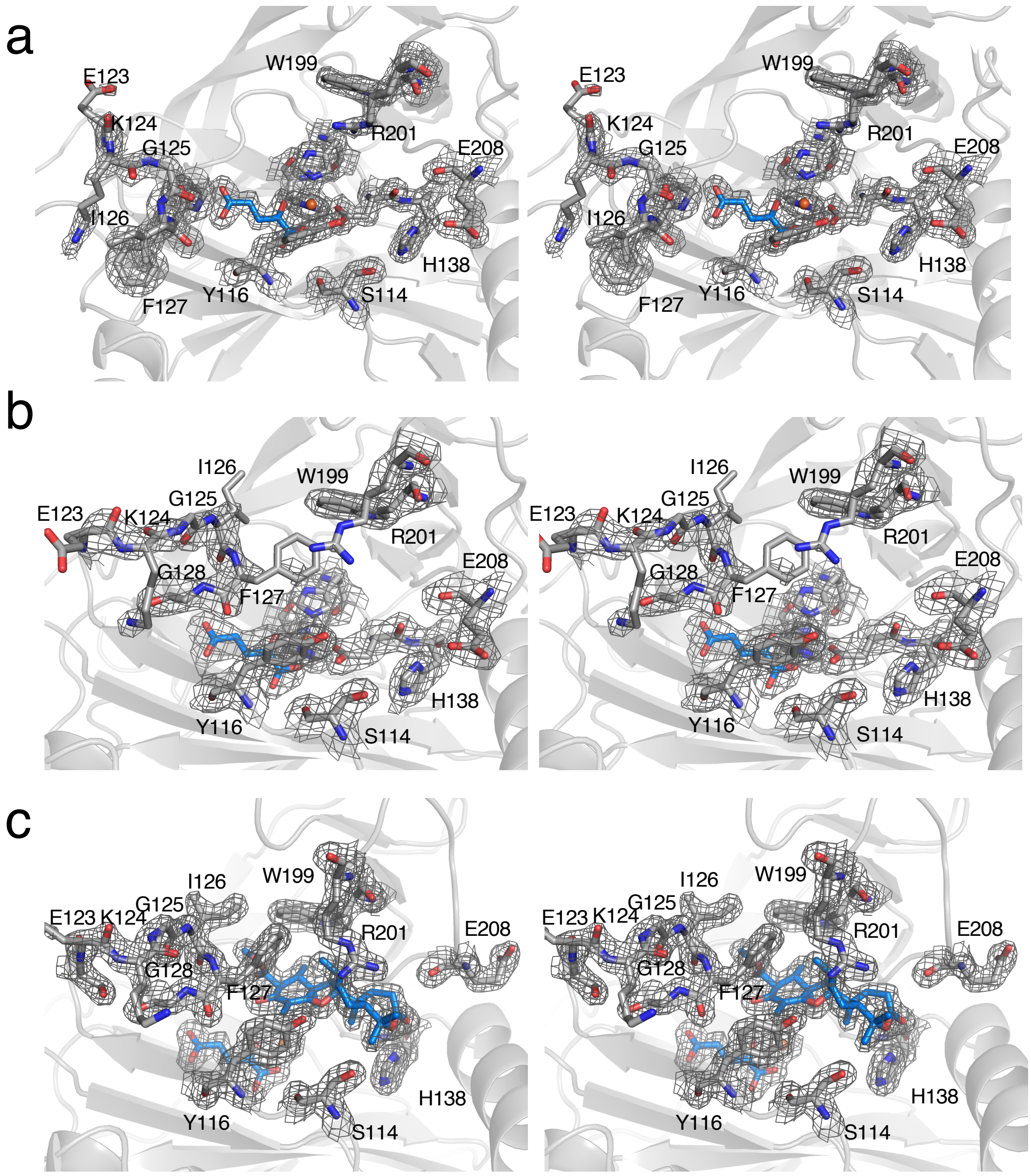

Supplementary Figure 7. Stereo views of active site architectures of NvfI. 2Fo-Fc maps of active site residues in (a) state I, (b) state II, and (c) state III. The electron density maps of active site residues and ligands are represented by a gray mesh, contoured at +1.0 sigma.

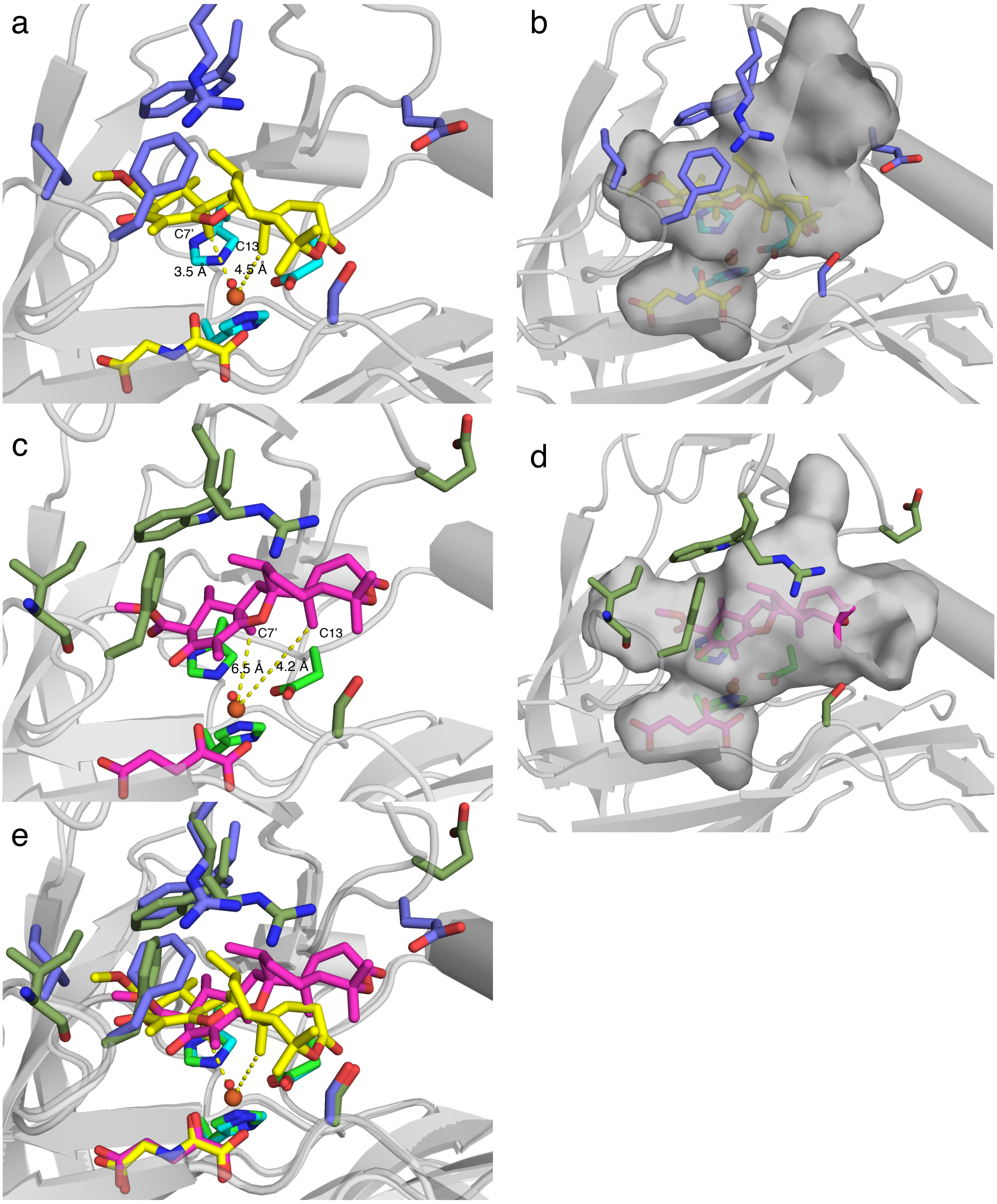

Supplementary Figure 8. Modeling of the substrate binding in the open conformation of NvfI. a) Model of the complex structure of NvfI (state II) with **2**. b) Surface view of the state II of NvfI in complex with **2** (model). c) The crystal structure of NvfI with **2**. d) Surface views of the crystal structure of NvfI in complex with **2**. e) Superimposed view of a) and c).

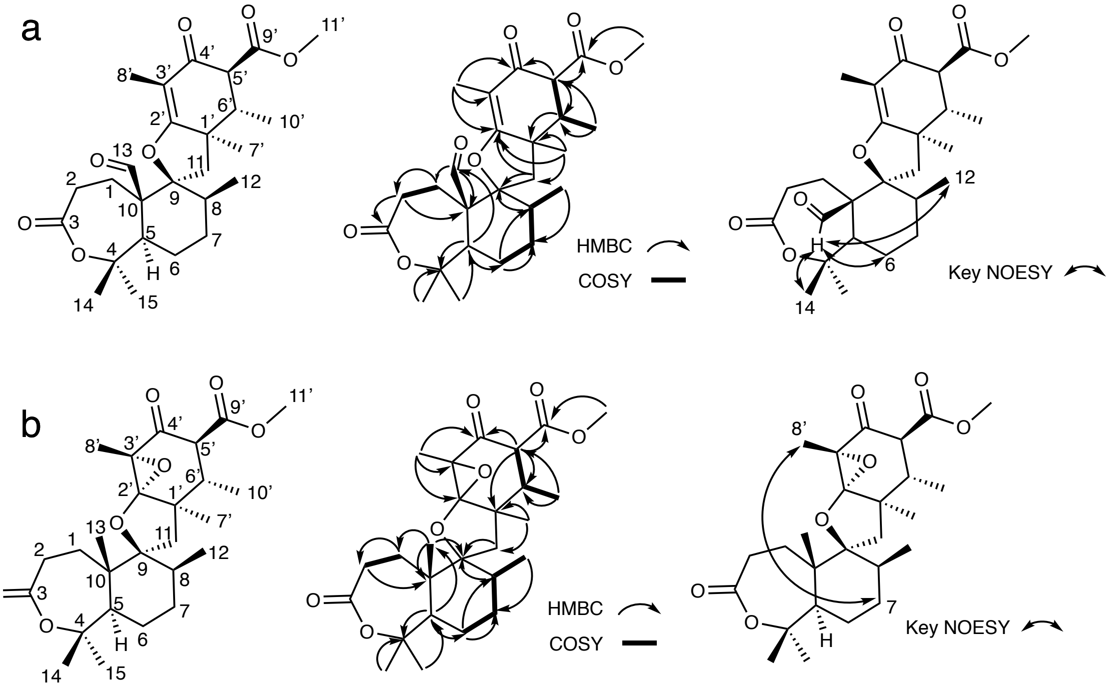

Supplementary Figure 9. Key HMBC, COSY, and NOESY correlations of (a) **5** and (b) **6**.

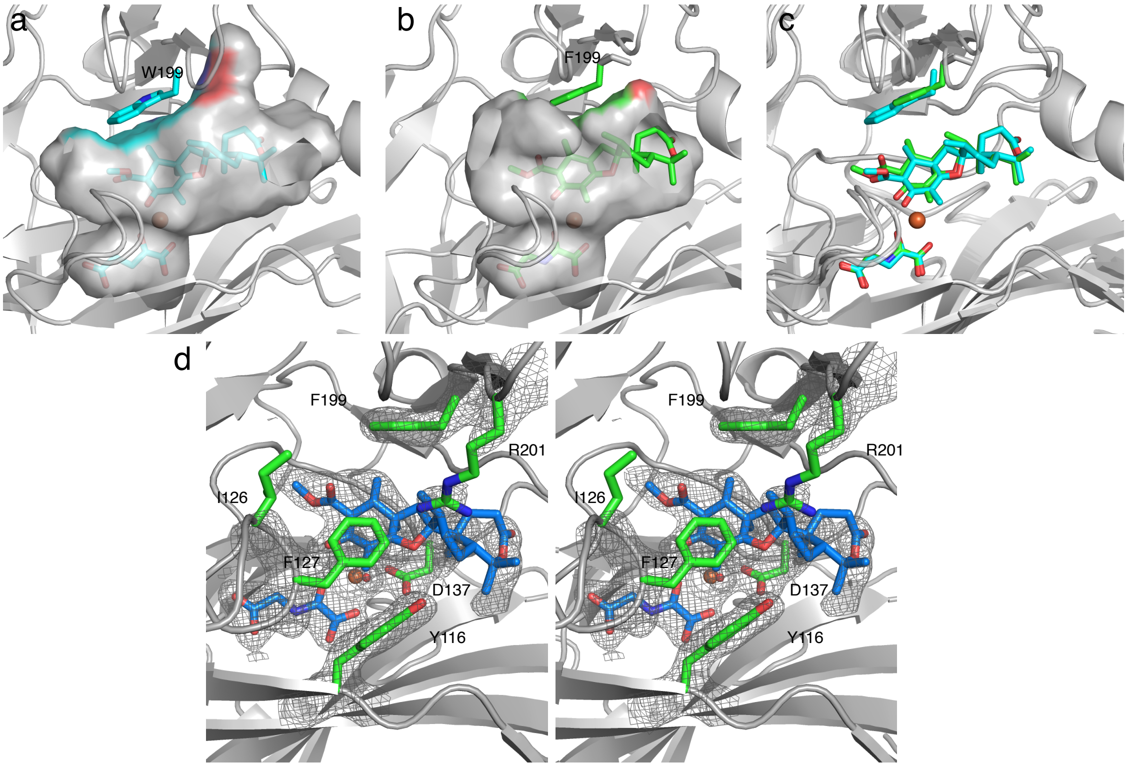

Supplementary Figure 10. Comparison of the active site of NvfI wild type and W199F variant. (a) The active site of wild type, (b) the active site of W199F, and (c) superimposed view of wild type and W199F variant. (d) Stereo views of active site architectures of NvfI W199F. Fo-Fc polder map of **2** and 2Fo-Fc maps of active site residues. The electron density maps of **2** (contoured at +3.0 sigma) active site residues (contoured at +1.0 sigma) are represented by a gray mesh.

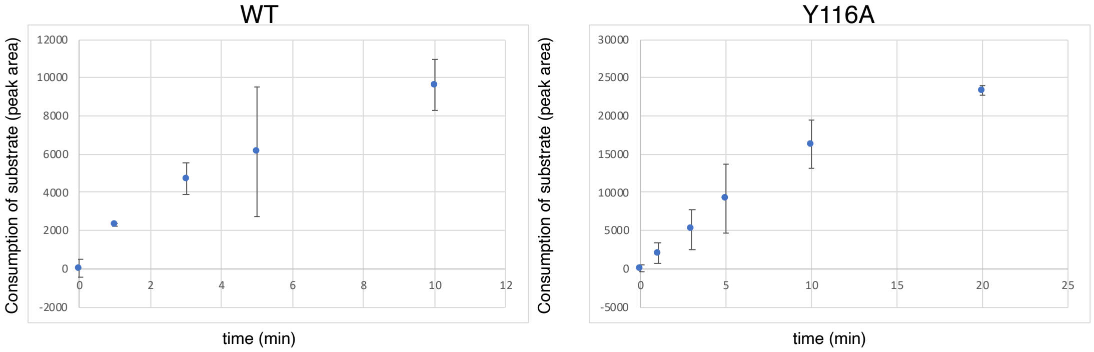

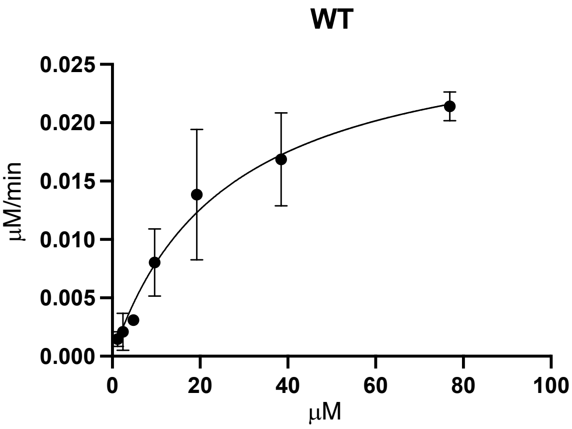

k_{cat}	0.29 ± 0.04 s^{-1}
K_M	25.9 ± 7.2 μM
k_{cat}/K_M	1.1×10^4 $M^{-1}S^{-1}$

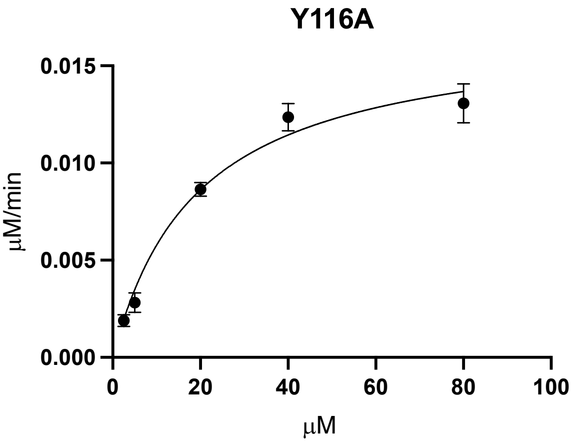

k_{cat}	0.17 ± 0.01 min^{-1}
K_M	19.5 ± 4.8 μM
k_{cat}/K_M	0.9×10^4 $M^{-1}S^{-1}$

Supplementary Figure 11. Time course enzyme reaction and Michaelis-Menten plots of the NvfI and NvfI-Y116A kinetics. These plots are means of triplicate experiments. Error bars, S.E.M.

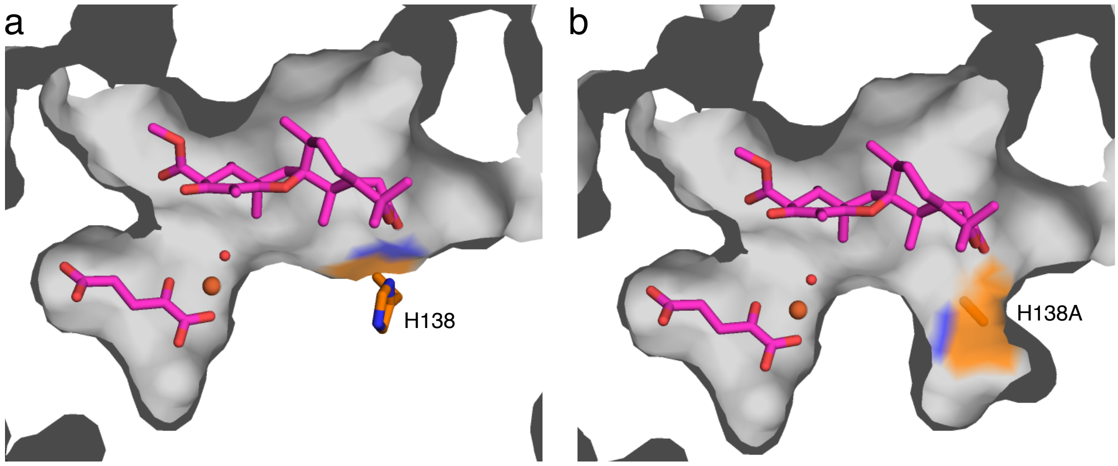

Supplementary Figure 12. The active site shapes of (a) NvfI wild type and (b) H138A variant (model).

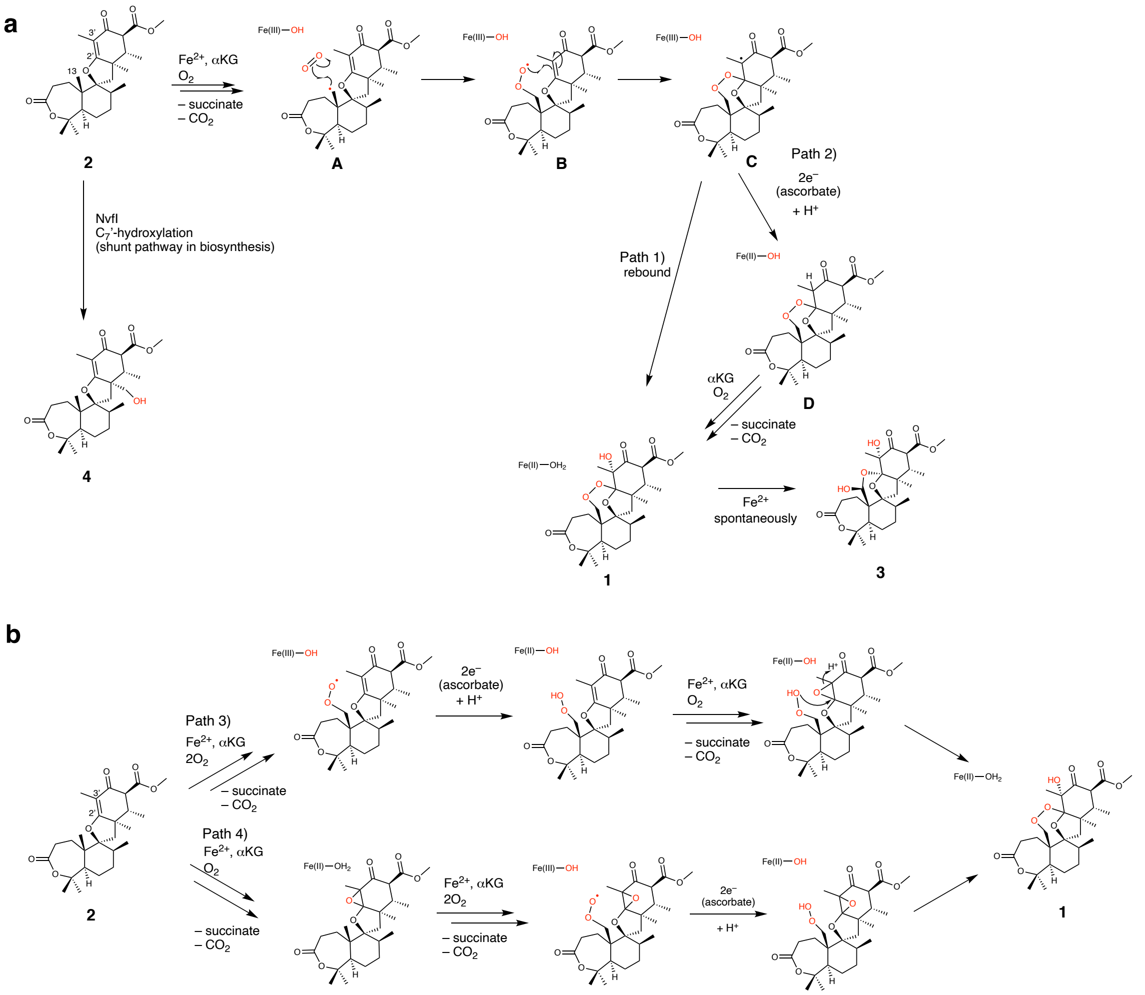

Supplementary Figure 13. The enzyme reaction of NvfI and possible reaction mechanism for the formation of **1**. (a) Proposed pathways 1 and 2 of NvfI. The generation of the C13 radical and the following endoperoxide formation produce the intermediate **C**. The oxygen rebound then occurs on the C3' radical to generate **1** (path 1) or alternatively, **C** may be reduced by a reductant (such as ascorbate under the *in vitro* conditions) to generate **D** (Path 2). The subsequent conversion of **D** to **1** would require another round of NvfI catalysis, which consumes another equivalent of both α-KG and O_2. (b) Proposed pathways 3 and 4 of NvfI. In path 3, **1** is generated *via* intermediate B, and the epoxide is formed on C2'-C3' of the peroxide intermediate. In path 4, the epoxide reaction occurs first, and then the peroxide-formation and cyclization reactions produce **1**.

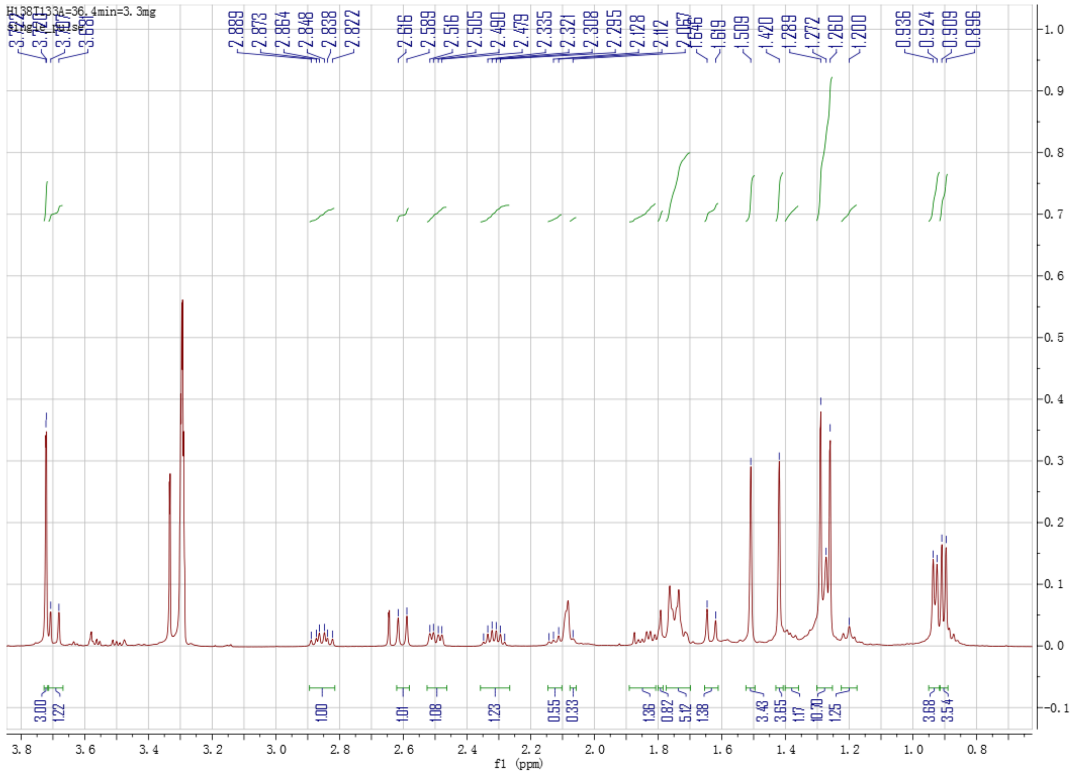

Supplementary Figure 14. ^{1}H NMR spectrum of **5** in methanol-d_4 (900 MHz).

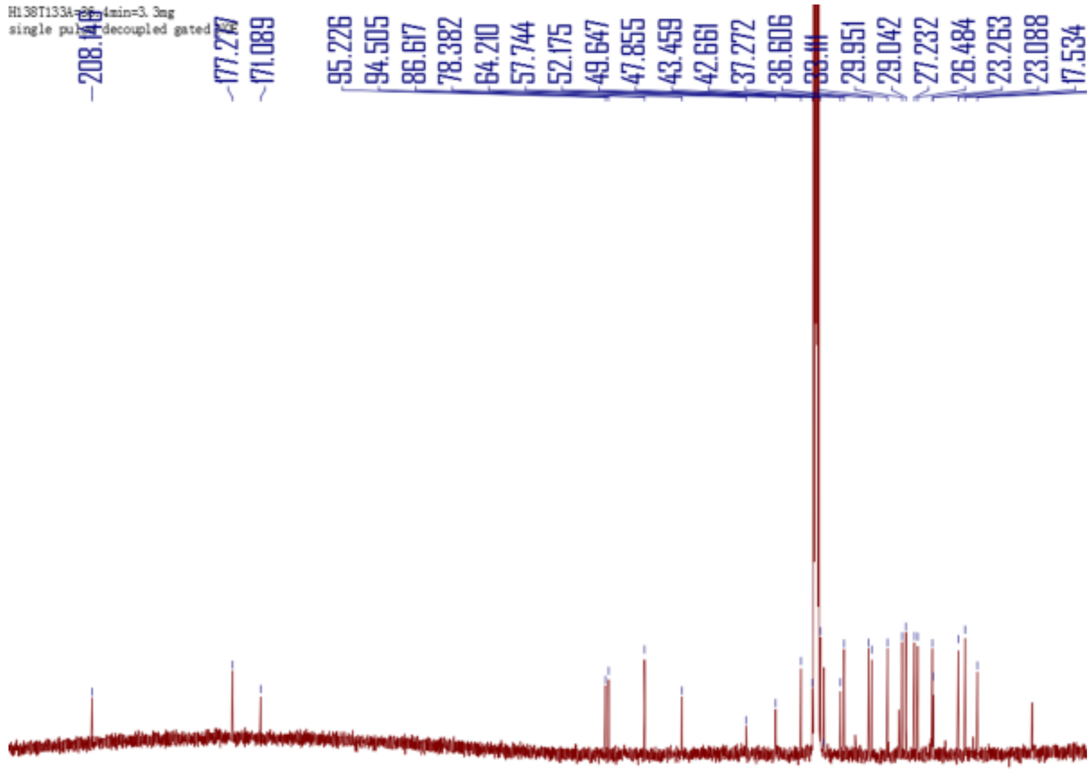

Supplementary Figure 15. ^{13}C NMR spectrum of **5** in methanol-d_4 (225 MHz).

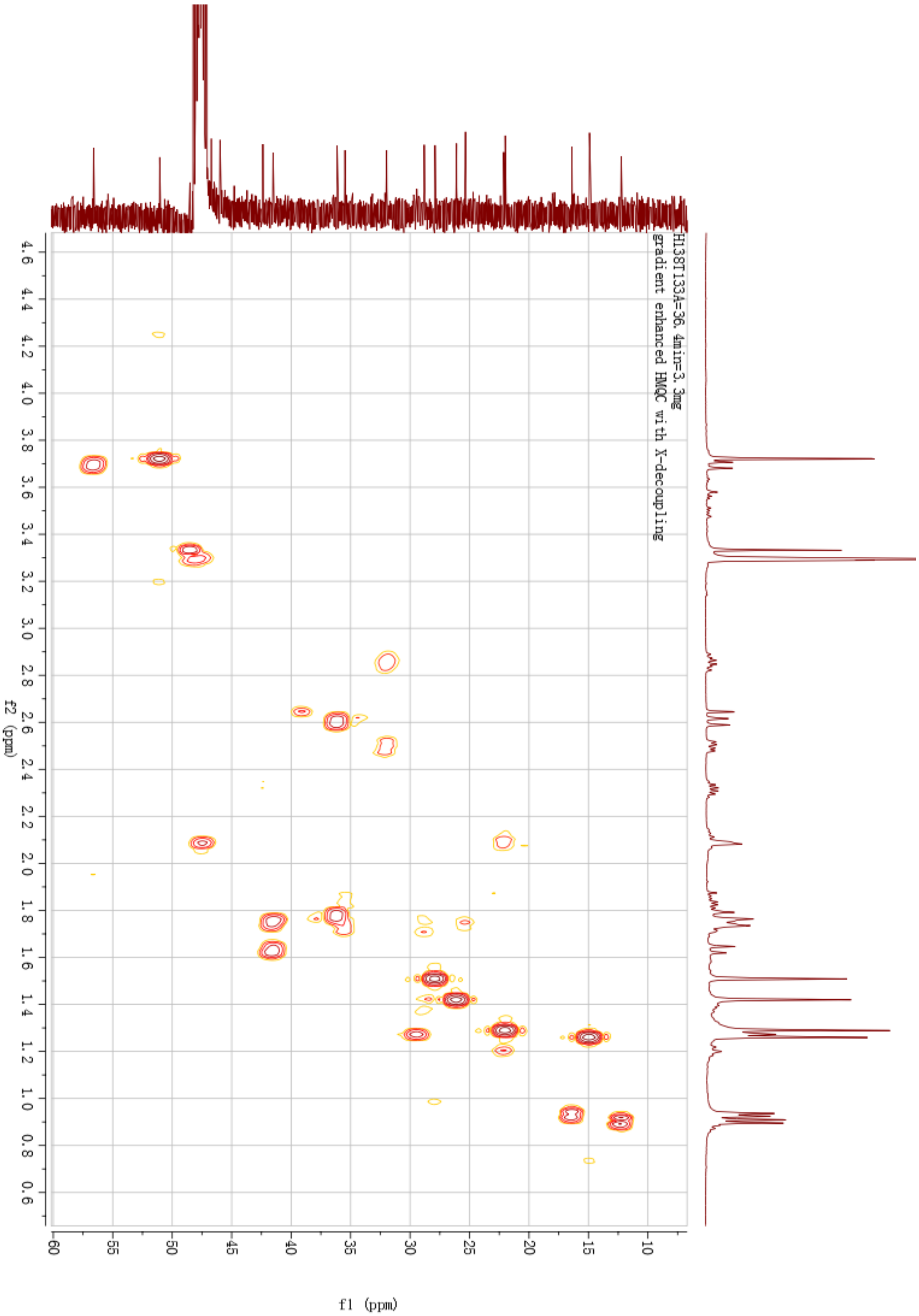

Supplementary Figure 16. HSQC spectrum of **5** in methanol-d_4 (225 MHz).

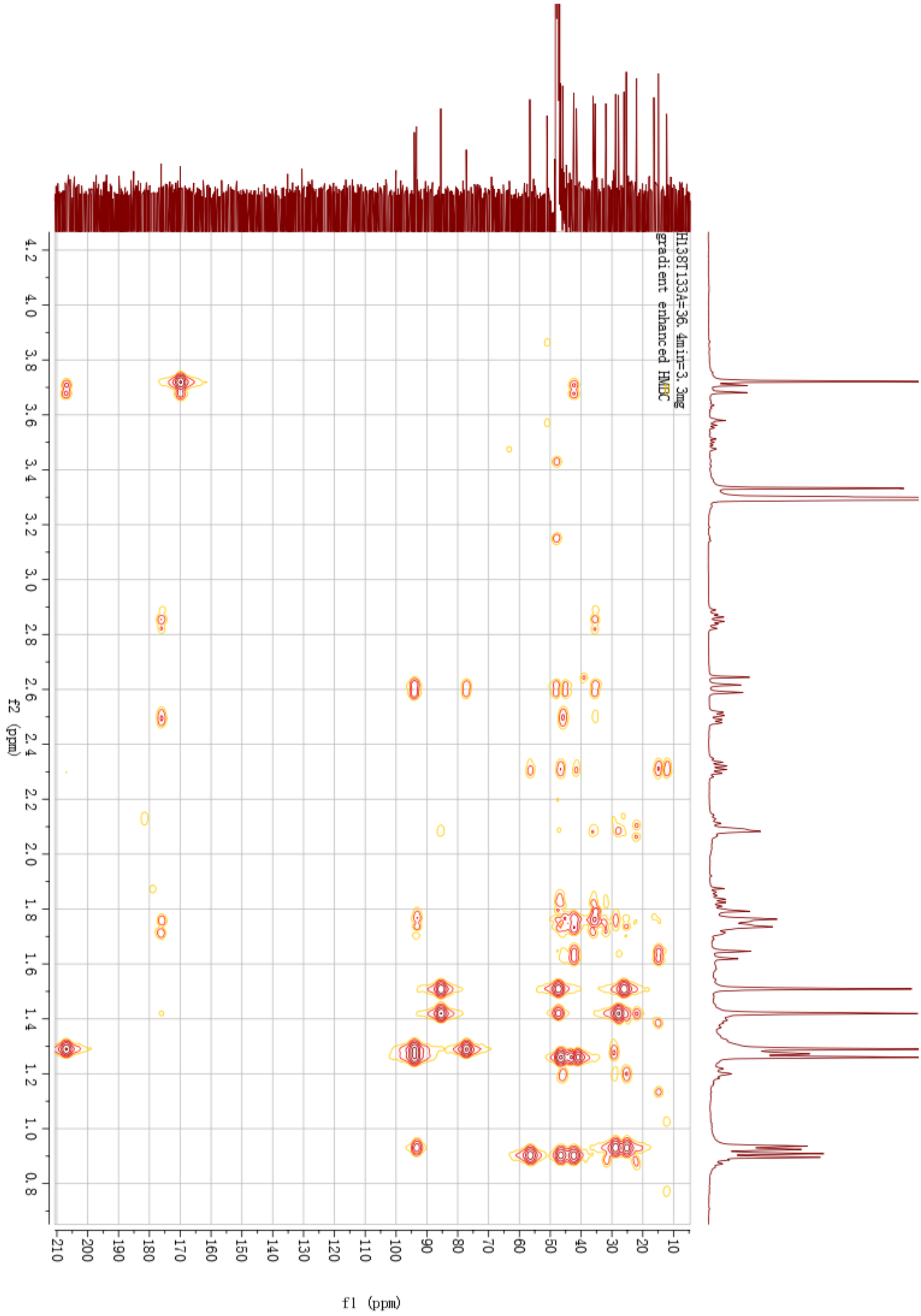

Supplementary Figure 17. HMBC spectrum of **5** in methanol-d_4 (225 MHz).

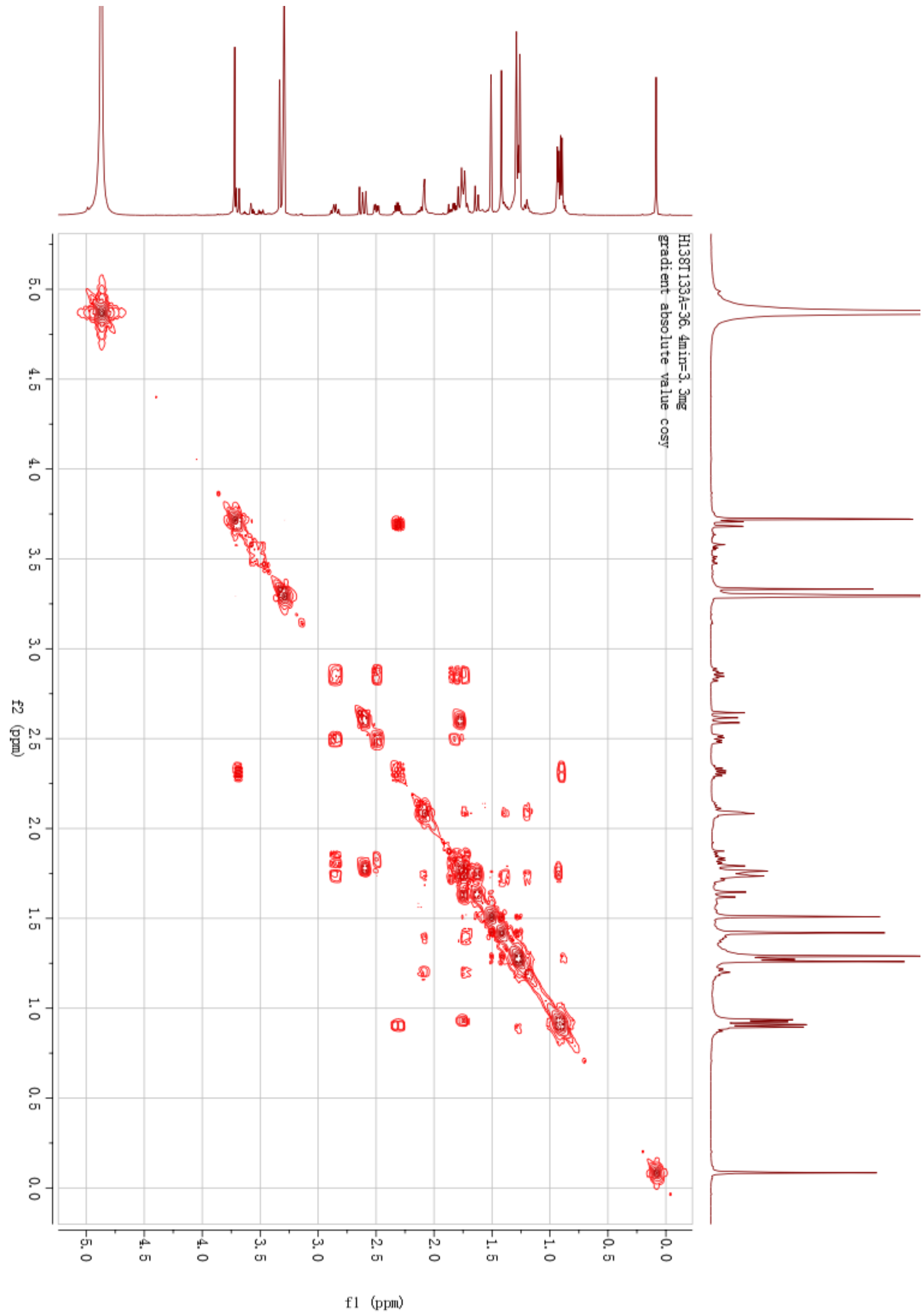

Supplementary Figure 18. COSY spectrum of **5** in methanol-d_4 (900 MHz).

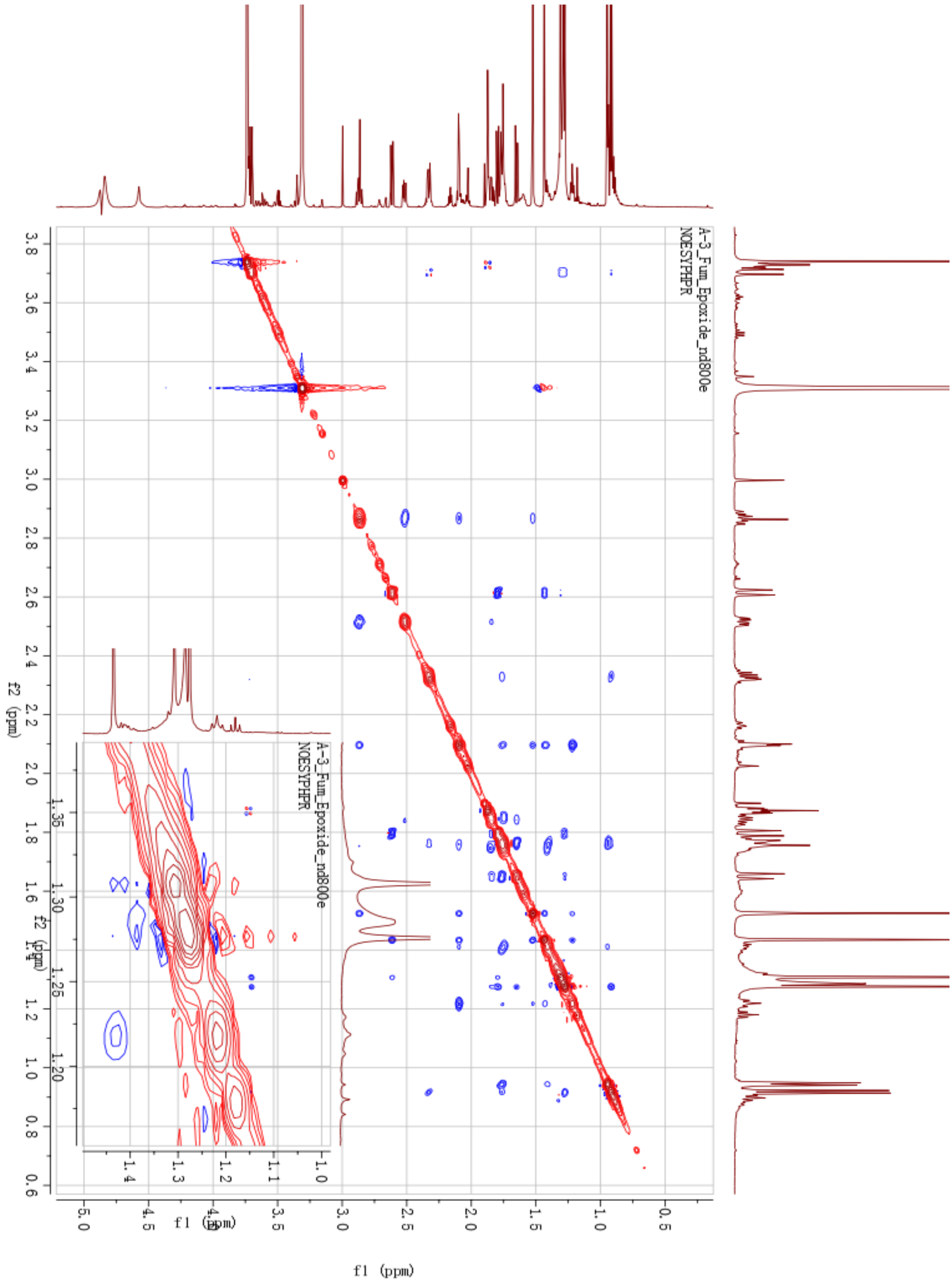

Supplementary Figure 19. NOESY spectrum of **5** in methanol-d_4 (900 MHz).

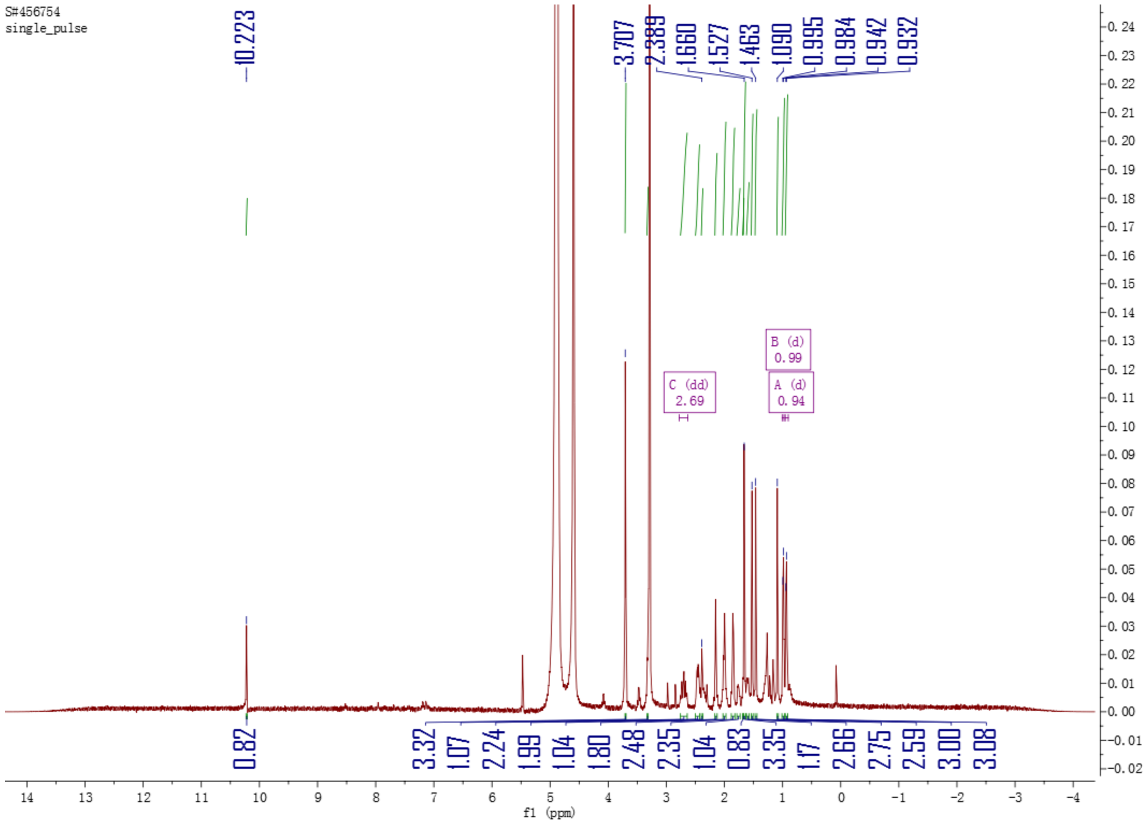

Supplementary Figure 20. ^{1}H NMR spectrum of **6** in methanol-d_4 (900 MHz).

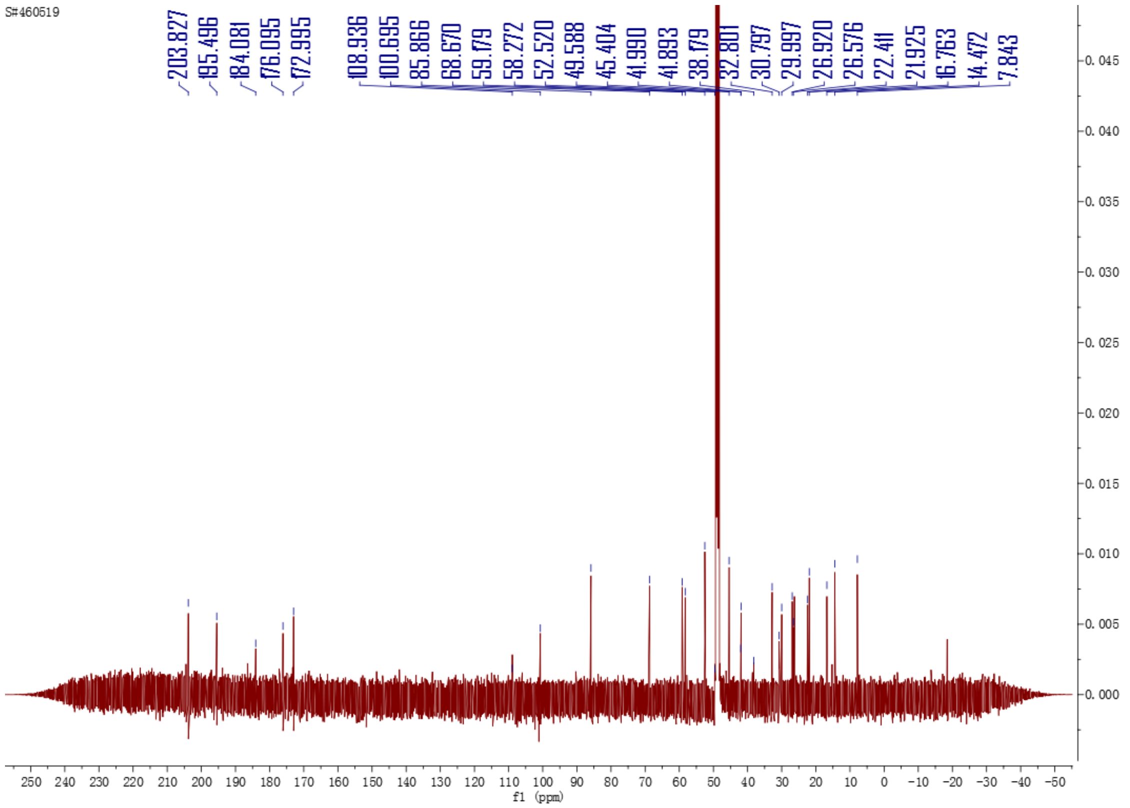

Supplementary Figure 21. ^{13}C NMR spectrum of **6** in methanol-d_4 (225 MHz).

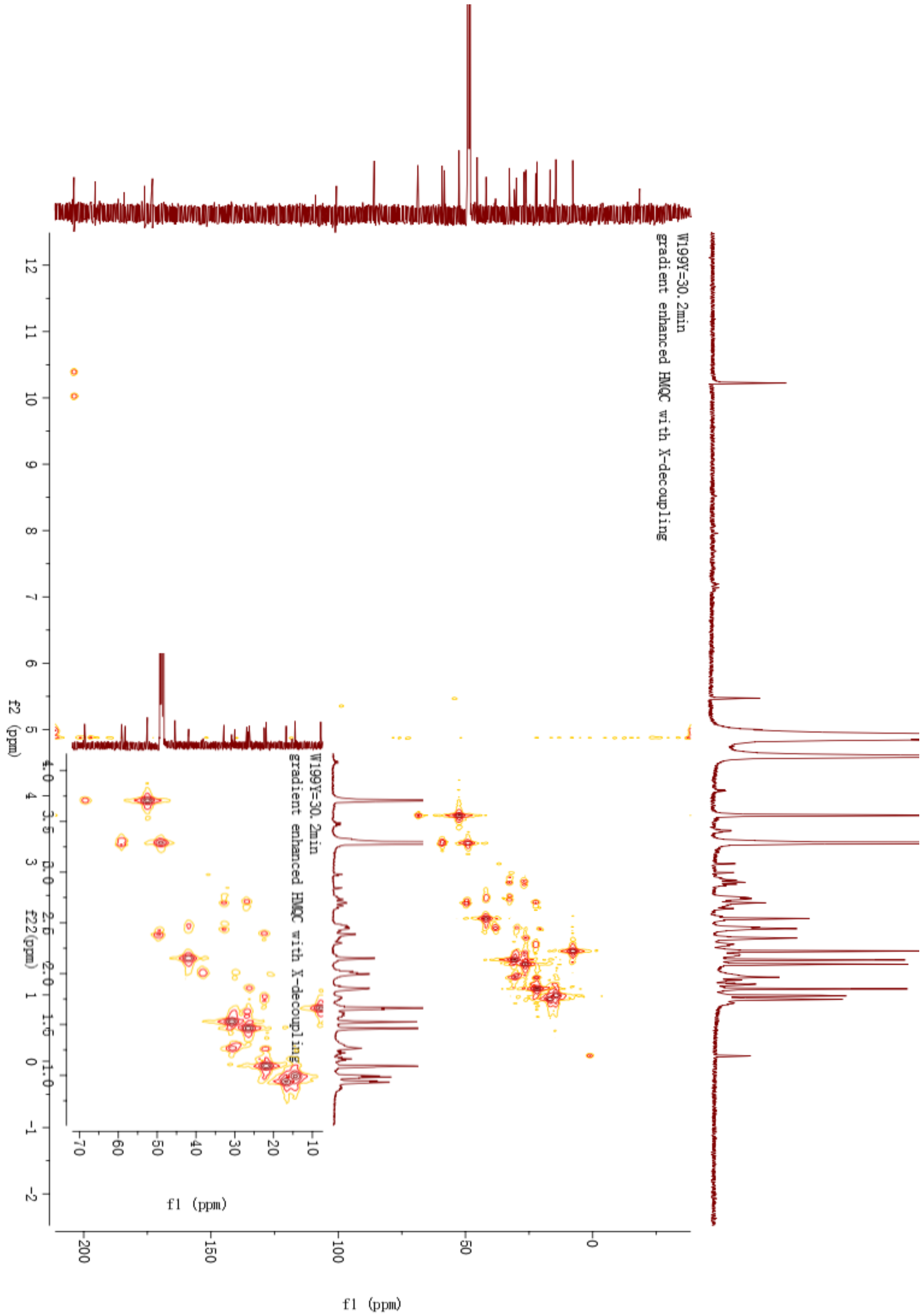

Supplementary Figure 22. HSQC spectrum of **5** in methanol-d_4 (225 MHz).

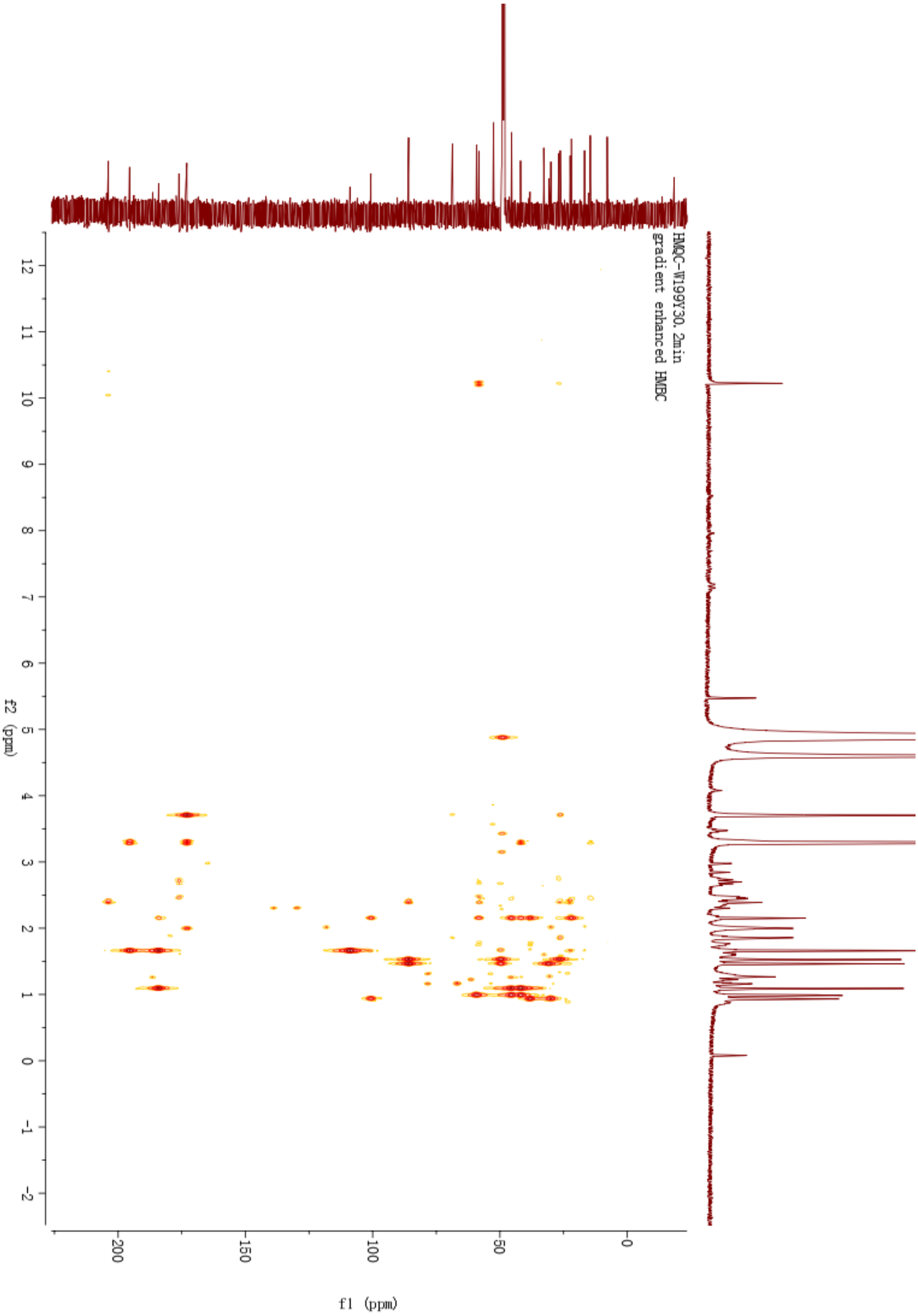

Supplementary Figure 23. HMBC spectrum of **6** in methanol-d_4 (225 MHz).

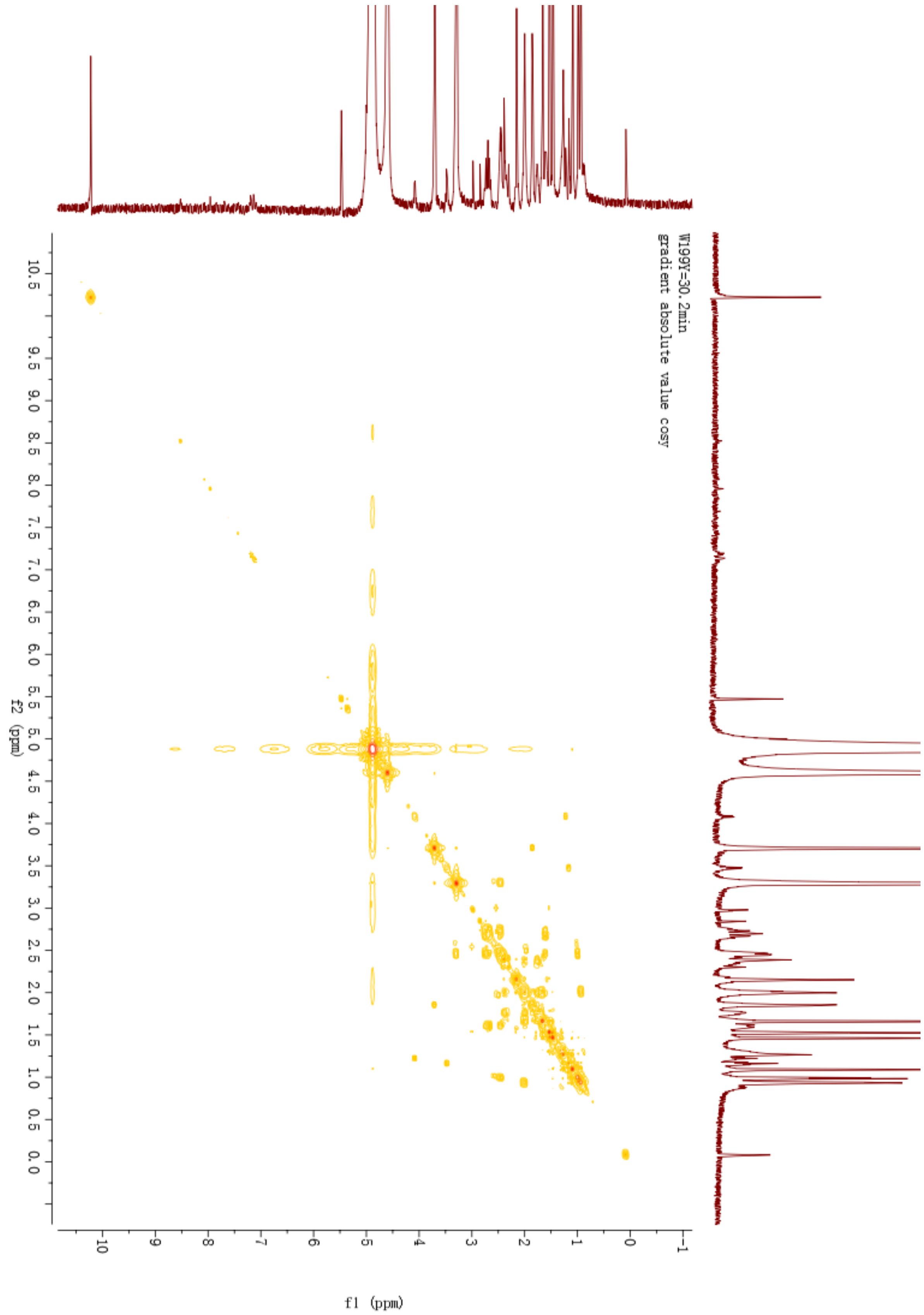

Supplementary Figure 24. COSY spectrum of **6** in methanol-d_4 (900 MHz).

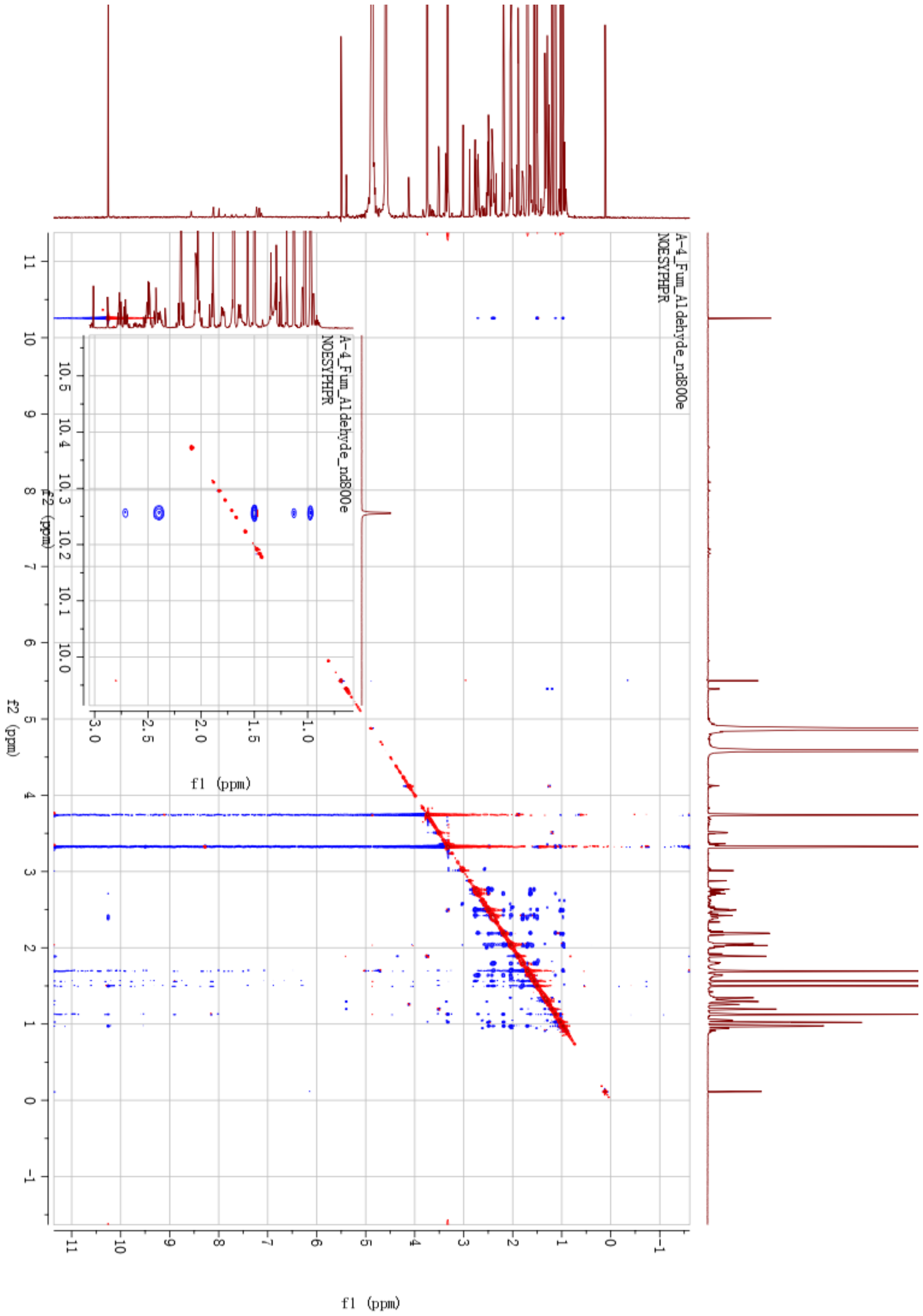

Supplementary Figure 25. NOESY spectrum of **6** in methanol-d_4 (900 MHz).
